# Synthesis and Optical Enhancement of Amorphous Carbon Nanotubes/Silver Nanohybrids via Chemical Route at Low Temperature

**DOI:** 10.1155/2014/847806

**Published:** 2014-06-05

**Authors:** Tan Kim Han, Leo Bey Fen, Ng Meng Nee, Mohd Rafie Johan

**Affiliations:** Nanomaterials Engineering Research Group and Advanced Materials Research Group, Department of Mechanical Engineering, University of Malaya, Lembah Pantai, 50603 Kuala Lumpur, Malaysia

## Abstract

We report the synthesis of amorphous carbon nanotubes/silver (**α**CNTs/Ag) nanohybrids via simple chemical route without additional reactant and surfactant at low temperature. Field emission scanning microscope (FESEM) and transmission electron microscope (TEM) confirmed formation of CNTs. X-ray diffraction (XRD) pattern confirmed the amorphous phase of carbon and the formation of Ag nanoparticles crystalline phase. Raman spectra revealed the amorphous nature of **α**CNTs. UV-visible spectroscopy showed enhancement of optical properties of **α**CNTs/Ag nanohybrids.

## 1. Introduction

Since the discovery of crystalline carbon nanotubes (CNTs) by Iijima [1], they have attracted worldwide interest due to their exceptional properties such as high mechanical strength, high electrical conductivity, and high thermal conductivity. Therefore, CNTs play a great role in a tremendously diverse range of research and application. However, crystalline CNTs pose a challenge for being produced in a large quantity due to their very critical deposition conditions like high operating temperature, expensive cost, long synthesis period, complex processing steps, and others [2]. In that light, amorphous carbon nanotubes (*α*CNTs) have become alternative to the crystalline CNTs due to their ease of production in large quantities [3]. *α*CNTs have unique amorphous structures, which are different from crystalline CNTs due to the wall composed of carbon clusters with a short-distance order or long-distance disorder [4]. The properties of CNTs are affected by their amorphous structural arrangement. They can be synthesized by arc discharge [5], chemical vapor deposition (CVD) [6], laser ablation [7], and chemical route [8].

Nevertheless, the *α*CNTs properties can be improved for various potential applications through hybridization of CNTs with noble metal such as silver [9], gold [10], and platinum [11] or with semiconductors such as cadmium selenide quantum dots [2]. The unique structure of CNTs enables them to be considered as a template for metal nanoparticles to form nanohybrids. In order to deposit nanoparticles on the inert CNTs wall, functional groups such as carboxylic acid, carbonyl, and hydroxyl groups need to be introduced onto the surfaces of CNTs by additional chemical treatment under acidic conditions. The functional groups created will give rise to the preferred nucleation site for metal deposition and also enhance the solubility of the CNT [12, 13]. Nanohybrids possess the combination of advantageous physical, chemical, and optical properties from both CNTs and metal nanoparticles. They could reveal the unexpected quantum effects and alter the band gaps of CNTs [2, 3]. CNTs/Ag nanohybrids received considerable attention due to the outstanding characteristic such as high catalyst activity [14], great optical properties [15], electrochemical sensor [16], and bactericidal properties in biomedical materials [17]. From the approaches reported, the CNTs/Ag nanohybrids can be produced by physical evaporation, solid state reaction, wet chemical reaction, and electroless deposition [18].

In this paper, we report a simple method for the synthesis of *α*CNTs and for the first time Ag nanoparticles attached on the *α*CNTs walls. The *α*CNTs surface was first purified and functionalized by strong acids; carboxyl groups were added on nanotubes wall to create reaction point between Ag nanoparticles. The acid treated nanotubes act as a template and starting point for the formation of nanohybrids. The interactions between *α*CNTs and Ag nanohybrids such as morphological, structural, elemental, and optical properties were investigated.

## 2. Experimental

### 2.1. Materials and Sample Preparation

Ferrocene, Fe(C_5_H_5_)_2_ (98%), and ammonium chloride (NH_4_Cl) were purchased from Acros Organics and Fisher Scientific, respectively. Hydrochloric acid, HCl (37%), was purchased from R&M Chemicals and silver nitrate (AgNO_3_) was purchased from Fisher Scientific. All chemicals were used without further purification.

*α*CNTs were prepared by mixing 2 g of Fe(C_5_H_5_)_2_ and 4 g of NH_4_Cl together in a covered crucible. The sample was heated in the furnace at 200°C for 30 min and allowed to cool at room temperature. The as-produced *α*CNTs were washed consecutively with diluted HCl and deionized water for several times and dried at 40°C for 24 h. The purified sample was functionalized by immersing in HNO_3_ at room temperature. The samples then were washed several times with deionized water and then dried at 40°C for 24 h. The functionalized *α*CNTs were hybridized with Ag nanoparticles.

100 mg of *α*CNTs was soaked in 200 mL deionized water for 5 min under ultrasonic dispersion. Different concentrations of AgNO_3_ (0.01 M, 0.05 M, 0.1 M, 0.5 M, and 1 M) were added into the *α*CNTs aqueous solution and continuously stirred at room temperature for 20 h. The product was filtered and washed with deionized water sequentially and dried at 40°C for 24 h. The nanohybrids functionalized by HCl and HNO_3_ were denoted as *α*CNTH/Ag and *α*CNTN/Ag, respectively.

### 2.2. Characterizations

The morphological structures of all samples were observed using field emission scanning electron microscope (FESEM, AURIGA Zeiss) and transmission electron microscope (TEM, Philips CM12) operated at 10 and 200 kV, respectively. Structural characterization was performed using X-ray diffractometer (XRD, Siemen D500) with Cu K*α* radiation (40 kV, 40 mA). The optical absorption spectra were recorded using UV/VIS spectrophotometry (Varian CARY 50 Series). Raman spectra for all samples were recorded using inVia Raman microscope (RENISHAW, United Kingdom).

## 3. Results and Discussion

**Figure 1:**
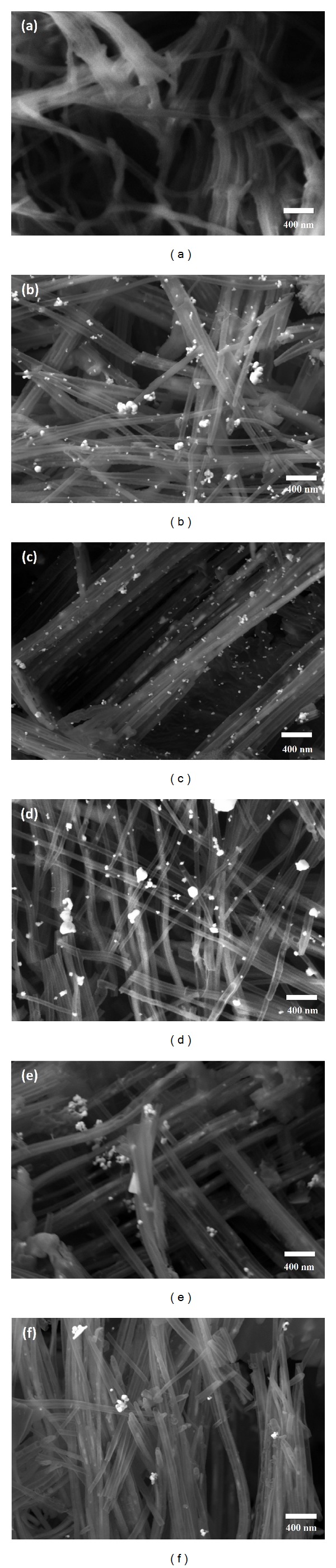
FESEM images of HCl functionalized *α*CNTH/Ag nanohybrids at different molars of AgNO_3_: (a) 0 M, (b) 0.01 M, (c) 0.05 M, (d) 0.1 M, (e) 0.5 M, and (f) 1 M.

**Figure 2:**
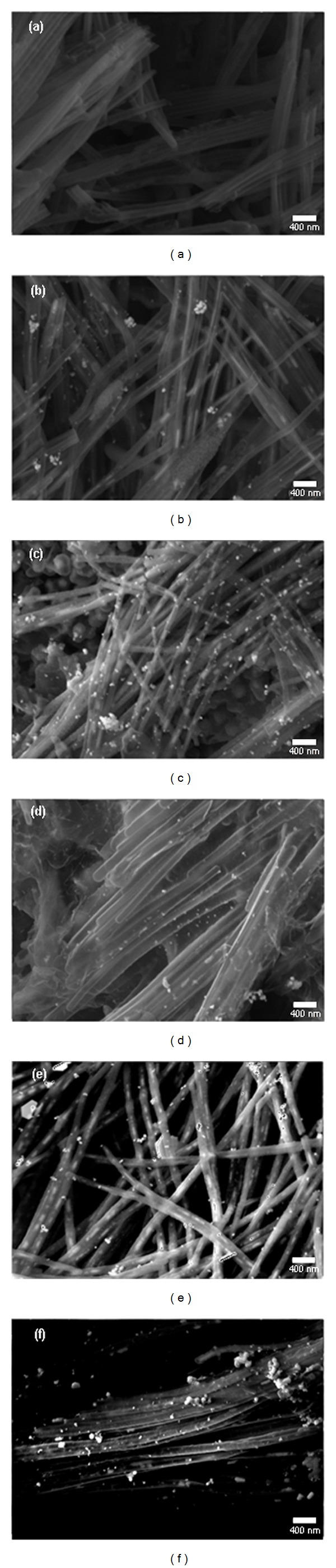
FESEM images of HNO_3_ functionalized *α*CNTN/Ag nanohybrids at different molars of AgNO_3_: (a) 0 M, (b) 0.01 M, (c) 0.05 M, (d) 0.1 M, (e) 0.5 M, and (f) 1 M.

The FESEM images of *α*CNTH/Ag and *α*CNTN/Ag nanohybrids at different molars of AgNO_3_ were shown in Figures 1 and 2, respectively. Figures 1(a) and 2(a) present FESEM images of the HCl and HNO_3_ functionalized *α*CNTs prior to hybridization. Both images show tubular structures and are present in a bundle form due to van der Waals forces. Meanwhile, Figures 1(b)–1(f) and 2(b)–2(f) show the FESEM images of hybridized HCl and HNO_3_ functionalized *α*CNTs at different molars of AgNO_3_. The walls of *α*CNT were randomly coated with Ag nanoparticles. The interaction sites of *α*CNT and Ag nanoparticles vary with increasing molarity of AgNO_3_. It was found that Ag nanoparticles tend to aggregate at higher molar of AgNO_3_.

**Figure 3:**
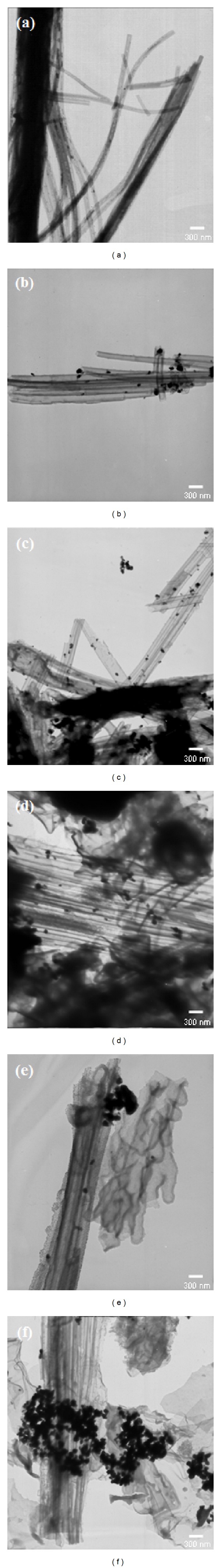
TEM images of HCl functionalized *α*CNTH/Ag nanohybrids at different molars of AgNO_3_: (a) 0 M, (b) 0.01 M, (c) 0.05 M, (d) 0.1 M, (e) 0.5 M, and (f) 1 M.

**Figure 4:**
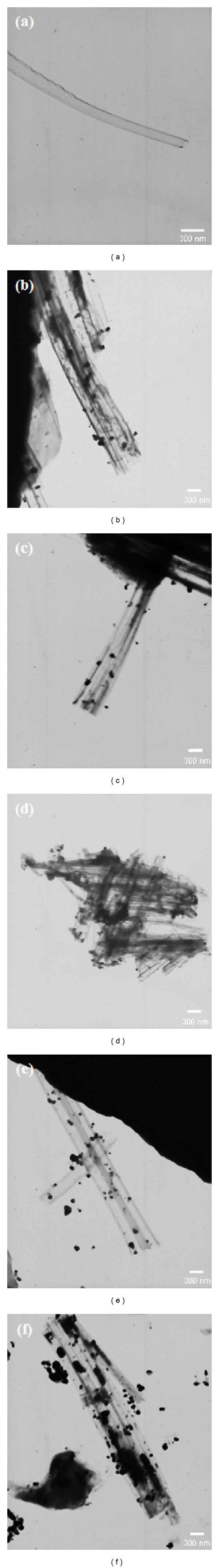
TEM images of HNO_3_ functionalized *α*CNTN/Ag nanohybrids at different molars of AgNO_3_: (a) 0 M, (b) 0.01 M, (c) 0.05 M, (d) 0.1 M, (e) 0.5 M, and (f) 1 M.

Figures 3 and 4 show the TEM images of HCl and HNO_3_ functionalized *α*CNTs/Ag at different molars of AgNO_3_. It is clearly observed that Ag nanoparticles are attached heterogeneously on the nanotubes wall. For HCl functionalized *α*CNTH/Ag, Ag nanoparticles are highly concentrated at specific sites on the CNTs wall at higher molar of AgNO_s_. Meanwhile, for HNO_3_ functionalized *α*CNTN/Ag, Ag nanoparticles are more scattered on the CNTs wall and not influenced by the various molars of AgNO_3_. This evidence confirmed that HNO_3_ manages to create more the reaction side than HCl.

**Figure 5:**
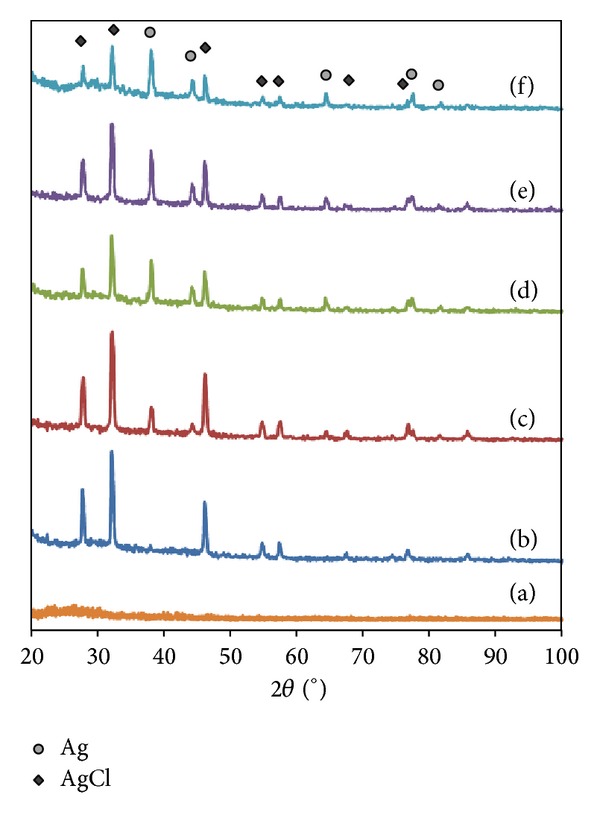
XRD patterns of HCl functionalized *α*CNTH/Ag nanohybrids at different molars of AgNO_3_: (a) 0 M, (b) 0.01 M, (c) 0.05 M, (d) 0.1 M, (e) 0.5 M, and (f) 1 M.

**Figure 6:**
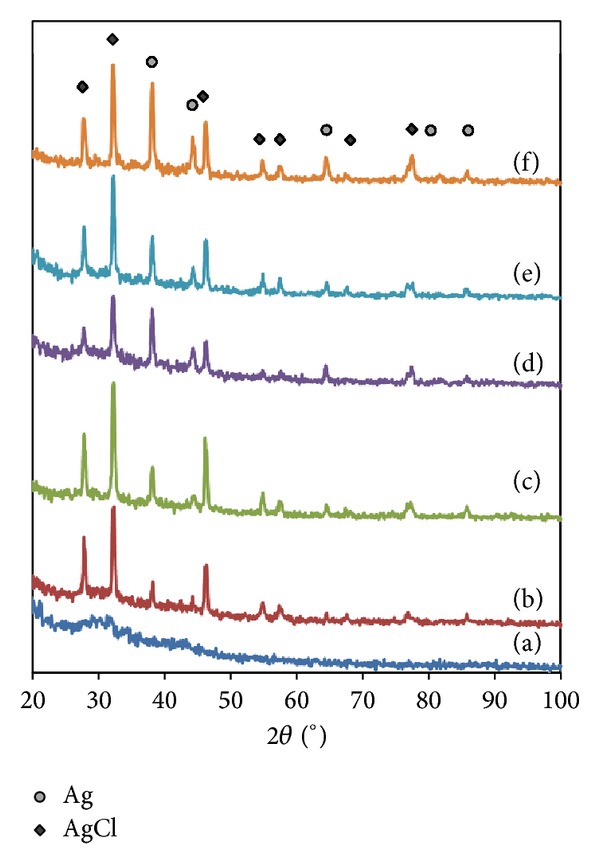
XRD pattern of HNO_3_ functionalized *α*CNTN/Ag nanohybrids at different molars of AgNO_3_: (a) 0 M, (b) 0.01 M, (c) 0.05 M, (d) 0.1 M, (e) 0.5 M, and (f) 1 M.

The XRD patterns of all samples obtained are illustrated in Figures 5-6. The as-synthesized *α*CNTs are amorphous with the broad peak at around 26° corresponding to (002) Bragg's reflections plane of graphite. The XRD patterns of the hybridized samples show diffraction peaks at angles of 38°, 44°, 64°, 77°, and 82° corresponding to (111), (200), (220), (311), and (222) planes of Ag nanoparticles structure. This result indicates that Ag nanoparticles are well crystallized on *α*CNTs wall. Meanwhile, additional peaks at angles of 28°, 32°, 46°, 55°, 58°, 68°, and 77° are also observed corresponding to the face-centered cubic (FCC) structure of AgCl. The formation of AgCl is resulted from the reaction of Ag^+^ with Cl^−^ during purification process [19]. However, AgCl peaks decrease significantly at higher molar of AgNO_3_. The trend is similar for HNO_3_ functionalized *α*CNTN/Ag nanohybrids.

**Figure 7:**
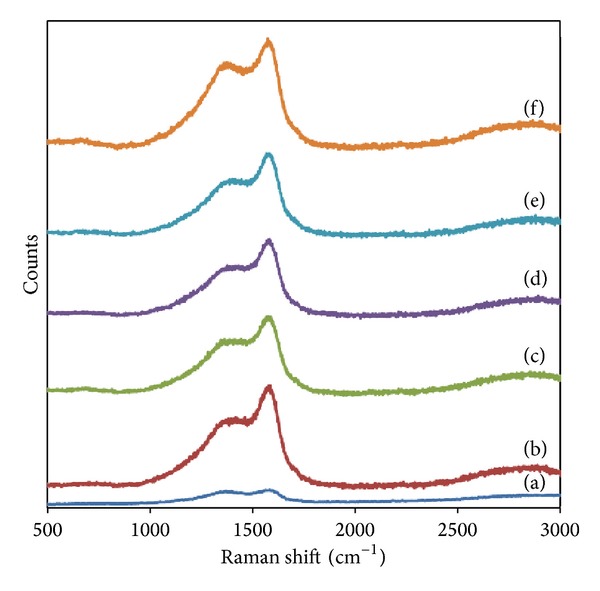
Raman spectra of HCl functionalized *α*CNTH/Ag nanohybrids at different molars of AgNO_3_: (a) 0 M, (b) 0.01 M, (c) 0.05 M, (d) 0.1 M, (e) 0.5 M, and (f) 1 M.

**Figure 8:**
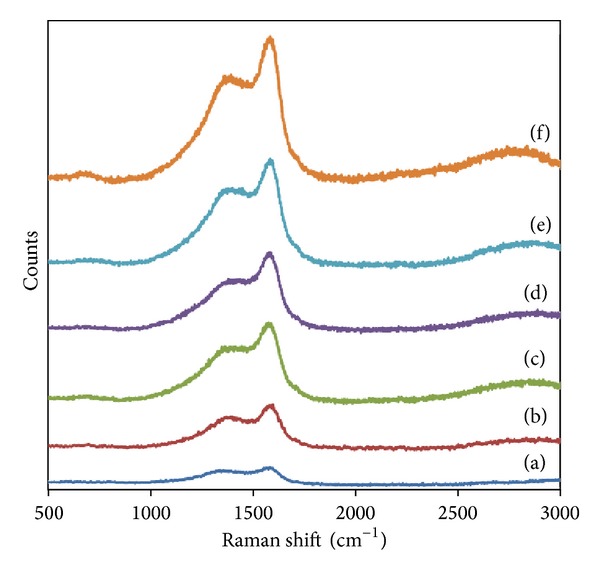
Raman spectra of HNO_3_ functionalized *α*CNTN/Ag nanohybrids at different molars of AgNO_3_: (a) 0 M, (b) 0.01 M, (c) 0.05 M, (d) 0.1 M, (e) 0.5 M, and (f) 1 M.

Figures 7-8 show the Raman spectra of HCl and HNO_3_ functionalized *α*CNTs/Ag nanohybrids at different molars of AgNO_3_ which are characterized by D and G bands at 1380 and 1583 cm^−1^, respectively. The G-band is attributed to the vibration of sp^2^ bonded carbon atoms [20] and D band is corresponding to the disordered carbon [21, 22]. The intensity of G (*I*_G_) band is increased significantly compared to D band (*I*_D_) for all molars of AgNO_3_. The similar trend is observed in HNO_3_ functionalized *α*CNTN/Ag nanohybrids. The decrease of *I*_D_/*I*_G_ ratio indicated the increase of crystallinity in nanohybrids' samples. The D and G bands are enhanced significantly after the deposition of Ag nanoparticles on *α*-CNTs. This can attribute to the plasmonic properties of Ag nanoparticles [23]. The depositions of Ag nanoparticles on *α*-CNTs wall directly affected the disorder degree in nanotubes due to the presence of crystalline Ag nanoparticles. This will enhance the G band significantly. The results are consistent with the previous XRD patterns.

**Figure 9:**
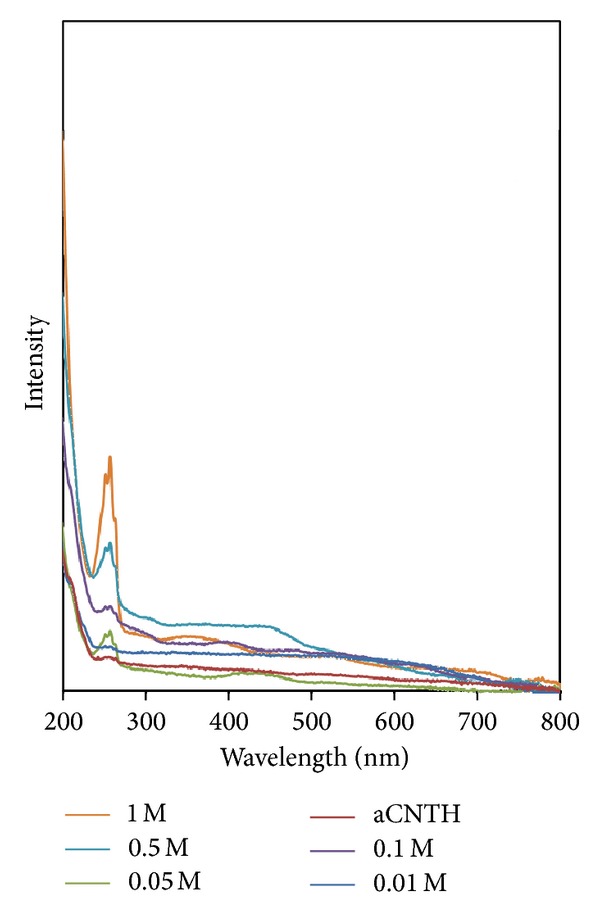
Absorption spectra of HCl functionalized *α*CNTH/Ag nanohybrids at different molars of AgNO_3_.

**Figure 10:**
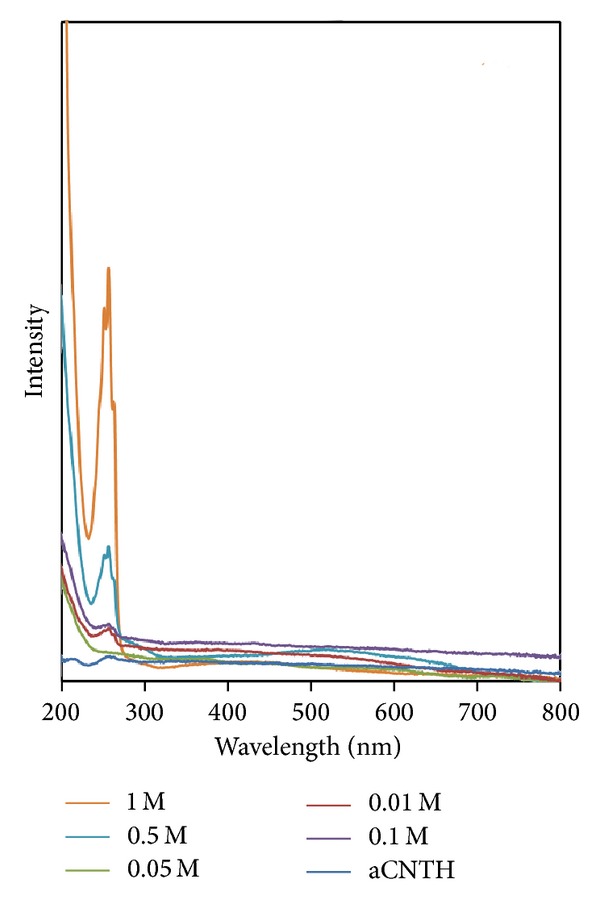
Absorption spectra of HNO_3_ functionalized *α*CNTN/Ag nanohybrids at different molars of AgNO_3_.

Figures 9-10 show the absorption spectra for all samples. The absorption peak at around 260 nm is corresponding to the *α*CNTs [24]. It was found that the absorbance increases with the increase of molarity of AgNO_3_. The linear increase of absorbance indicates that the Ag nanoparticles are well bonded on nanotubes' walls [25]. In addition, there is another band observed in the visible region at 440 nm. This excitonic feature indicates a monodispersed collection of Ag nanoparticles in the nanotubes during hybridization. Thus, the introduction of Ag nanoparticles had relatively enhanced the optical properties of *α*CNTs [15, 26].

**Figure 11:**
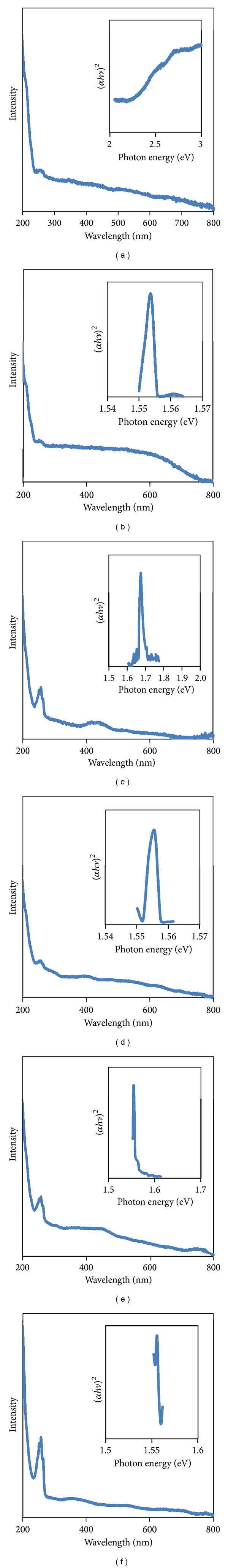
Optical absorbance spectra of HCl functionalized *α*CNTH/Ag nanohybrids at different molars of AgNO_3_: (a) 0 M, (b) 0.01 M, (c) 0.05 M, (d) 0.1 M, (e) 0.5 M, and (f) 1 M. Inset shows Tauc plot for each spectrum.

**Figure 12:**
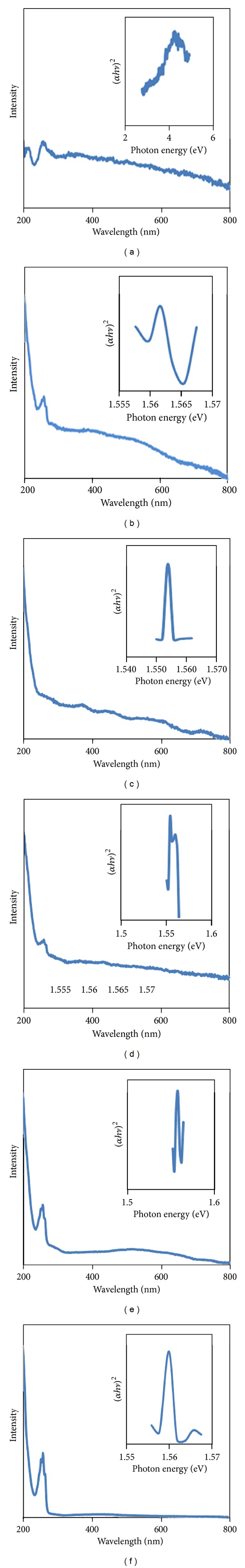
Optical absorbance spectra of functionalized *α*CNTN/Ag nanohybrids at different molars of AgNO_3_: (a) 0 M, (b) 0.01 M, (c) 0.05 M, (d) 0.1 M, (e) 0.5 M, and (f) 1 M. Inset shows Tauc plot for each spectrum.

The optical band gap can be evaluated using the Tauc relation [27, 28]. Consider

(1) $$\begin{array}{c} \left(\varepsilon h\nu \right)=C{\left(h\nu -E_{g}\right)}^{n}, \end{array}$$

where *C* is a constant, *ε* is a molar extinction coefficient, *E*_*g*_ is the optical band gap of material, and *n* depends on the type of transition. Figures 11-12 show the *E*_*g*_ values obtained from the interception of linear portion in the Tauc plots [28].  Table 1 summarizes the details of *E*_*g*_ values. *E*_*g*_ decreases significantly after the attachment of Ag nanoparticles on nanotubes wall. However, the increase molarity of AgNO_3_ did not further affect the band gap energy. Thus, the attachment of Ag nanoparticles on the nanotubes wall had enhanced the electrical conductivity of the *α*CNTs.

## 4. Conclusions

*α*CNTs/Ag nanohybrids were successfully synthesized by using a simple chemical technique. FESEM and TEM images showed that Ag nanoparticles were successfully anchored on the nanotubes' walls. They were found to have the tendency to agglomerate at a higher Ag concentration especially for HCl functionalized *α*CNTH/Ag nanohybrids. The peaks of newly introduced Ag crystalline structure were detected in XRD spectra. Besides that, peaks which refer to AgCl were also found in the nanohybrids system. The attachment of Ag nanoparticles on nanotubes wall enhanced the G band in Raman spectra significantly. As a result, *I*_D_/*I*_G_ ratio was gradually decreased, indicating a higher crystallinity degree in nanohybrids samples. The attachment of Ag nanoparticles on *α*CNTs was found to enhance their optical properties. The optical band gap energy of nanohybrids decreased after the loading of Ag nanoparticles which prove that the Ag nanoparticles had improved the conductivity properties of *α*CNTs themselves. These unique properties from *α*CNTs/Ag nanohybrids system may find their advantages and usefulness for potential applications in various fields, such as medical [29], electronic [30], and waste water treatment [31].

## Figures and Tables

**Table 1:** The optical band gap energy (*E*_*g*_) of all samples HNO_3_ purified *α*CNTN/Ag nanohybrids.

Samples at different molars of AgNO_3_ (M)	Optical band gap energy (eV)	
	HCl functionalized *α*-CNTN/Ag	HNO_3_ functionalized *α*-CNTN/Ag
0.00	2.09	2.30
0.01	1.55	1.56
0.05	1.65	1.55
0.10	1.55	1.55
0.50	1.55	1.55
1.00	1.55	1.56
